# Pectinolytic and Cellulolytic Enzymes Enhance *Fusarium compactum* Virulence on Tubercles Infection of Egyptian Broomrape

**DOI:** 10.1155/2010/273264

**Published:** 2011-01-16

**Authors:** Olubukola O. Babalola

**Affiliations:** Department of Biological Sciences, Faculty of Agriculture, Science and Technology, North-West University, Mafikeng Campus, Private Bag X2046, Mmabatho 2735, South Africa

## Abstract

The use of enzyme could facilitate pathogen penetration into plant host. Here the combination of cellulase and pectinase was ascertained on the pathogenicity of *F. compactum* (1.4 × 10^6^  propagules ml^−1^) on broomrape tubercles. *F. compactum* alone infected all the inoculated tubercles but did not kill any significant number. Infested tomato roots that were inoculated with mycelia plus pectinase (20 U ml^−1^) had over 50% tubercles dead one week after treatment. Those inoculated with mycelia plus cellulase (20 U ml^−1^) had above 60% mortality. Mixtures of mycelial plus the two enzymes (10 U ml^−1^ of each enzyme) showed synergy. The activity catalyzed by an enzyme is a measure of the amount of enzyme present. It was shown that, in a 1 mg (10 U mg^−1^) cellulase used, 0.055 mg pectinase (1.1 U mg^−1^) is present. This explains why mycelial plus cellulase mix contends with mycelial plus the two enzymes.

## 1. Introduction

Broomrapes (*Orobanche* spp.) are fully parasitic flowering plants that lack chlorophyll; hence they penetrate roots of susceptible hosts, removing water, minerals and sugars. *Orobanche aegyptiaca* Pers. (Egyptian broomrape) attacks dicotyledonous crops cultivated around the Mediterranean, causing massive yield losses [1]. Broomrape attached to the host by means of tubercle, a swollen organ which may be simple or composite. With the exception of the case of transgenic target-site, herbicide-resistant host plants, meant to be a temporary measure until other effective control means are found [2]. *Orobanche* generally maintains a close relationship with the host and so it is unreasonable to attack it using herbicides because the latter may adversely affect the nontarget host. Despite research on *Orobanche* spp. for over three decades, yield losses still abound because there is no sustainable method for controlling the parasite. 

*Fusarium compactum* (Wollenw.) Gordon, used in this study is a pathogen of broomrape plants. The inundative biocontrol approach, with repeated applications of the biocontrol agent, generates a state of equilibrium with a very low level of weed density as a result of the artificial inoculation of the biocontrol agent but the fungi are not sufficiently virulent for field release, regardless of the amount used. A series of experiments were conducted using *F. compactum*, a biological control agent that infects *Orobanche* without affecting the roots of tomato.

Some fungi are reported to have pectinolytic and/or cellulolytic enzymes [3]. Pectins are complex polysaccharides and are one of the major components of the plant cell wall of dicotyledonous plants, where they control the ionic status, cell expansion, and separation [4]. Some cellulase producing fungi includes *Acremonium* sp, *Aspergillus* spp and *Fusarium* sp, [5], *Trichoderma* spp. [6, 7], *Zymomonas* [8], and mutant Penicillium [9]. In this paper, the cellulase used is a product of *Trichoderma viride*. A hydrolytic enzyme plays an important role in the pathogenicity of plants by facilitating fungal penetration through the host cell wall [10, 11]. Experiments using mycoherbicidal organisms plus pectinase (EC 3.2.1.15) [12] or cellulase (EC 3.2.1.4) [13] indicate that enzyme enhances the weed control of pathogenic fungi. Here, pectinolytic and cellulolytic enzymes have been used to enhance the virulence of *F. compactum* on tomato plants infested with broomrape. A semiaxenic polyethylene bag system was used that allowed easy visual observation of the fungal infection of the tubercles. This study reports that the addition of pectinase and cellulase alone or in mixtures enhanced the virulence of *F. compactum* on broomrape.

## 2. Materials and Methods

### 2.1. Pathogen Production

*F. compactum* was cultured on potato glucose agar (PDA, Pronadisa) in Petri dishes incubated at 25°C. Subcultures were grown in 100 ml potato glucose broth (PDB, Pronadisa) in 250 ml Erlenmeyer flasks. Chloramphenicol (50 mg l^−1^) was added to the broth to restrict possible bacterial contamination. The cultures were left on a rotary shaker (Brunswick Scientific) at 150 rpm for 48 h. *F. compactum* mycelia were harvested on Miracloth (Calbiochem, La Jolla, CA), rinsed with distilled water to remove remaining spores and excess medium, and harvested by vacuum filtration. The washed hyphae were chopped at 6,000 rpm for 2 min with a homogenizer (IKA T18 basic Ultra-Turrax USA), resuspended in sterile water, and the propagule concentrations of chopped mycelia were estimated after serial dilution and plating.

### 2.2. Broomrape Seeds and Tomato Seedlings

Broomrape seeds were kindly provided by Dr. J. Hershenhorn of the Newe Yaar Research Center (Ramat Ishay, Israel). Surface sterilizing seeds ensure that the fungal infection on the seeds is from deliberate infection. Thus, about 13 mg seeds in small bags formed of Miracloth were wetted and surface sterilized in 80% ethanol for 1 min and in a mixture of 1% sodium hypochlorite in 0.01% aqueous Tween 20 for 10 min. The seeds were rinsed three times in sterile distilled water. Tomato transplant plugs at the two to three leaf stages in Speedling Insert Trays were purchased from Hishtil Inc, Ashkelon, Israel. The seedlings were cultured for later use as host plants in the broomrape studies.

### 2.3. Growth Chamber Experiments

The pathogenicity of *F. compactum* was tested in the semiaxenic polyethylene bag system; briefly about 13 mg of dry surface-disinfected seeds (up to 1,500) were sprinkled on wet Whatman GF/A glass-fiber sheets (Whatman Int. Ltd., Maidstone, England) in each bag. The broomrape seeds were conditioned for a 7 d period on the wet glass-fiber sheets. A tomato seedling with three or four expanded leaves and washed roots was fixed inside each polyethylene bag containing conditioned broomrape seeds. The plant roots in each bag were moistened by capillary action with forty ml of modified Hoagland's solution in the base of each bag [14]. Modified Hoagland's solution was replenished as needed. The polyethylene bags were then hung on metal frames wrapped with black plastic. Tomato plants were grown at a constant temperature of 25°C in a growth chamber. Fourteen-hour photoperiods were provided by a photosynthetically active light intensity of 65 *μ*E/m^2^/s (LI-COR, Inc., photometer, Model LI-188B) produced by six 40 W cool white fluorescent tubes suspended 35 cm above the benches. Two ml of 5-*μ*g ml^−1^ GR-24 (synthetic germination stimulant) were added to each bag with a pipette to augment the tomato root exudates. This spreads by capillary action. The broomrape seeds germinated, attached to tomato roots, and formed small tubercles during the following 2 weeks. 

 Allocation of treatment to *Orobanche*-infested tomato plants were in such a way that the tubercle numbers and sizes were almost the same. The virulence of the fungus was determined with and without various concentrations of either pectinase (ex fungal origin, 1.1 U mg^−1^, Sigma) and/or cellulase (Cellulysin, ex *Trichoderma viride*, 10 U mg^−1^ Calbiochem-Behring Corp., La Jolla, CA 92037). The effect of cellulase concentration (10 to 20 U ml^−1^) on tubercle death was similarly determined at a constant inoculum level. Thereafter, the virulence of the fungus with the two enzymes was determined in combination at varying ratios. Control plants were mock-inoculated with either sterile distilled water containing 0.01% Tween 20 or 4 to 20 U ml^−1^ of single or combined enzyme preparations but without fungal mycelia. Tubercles on the tomato plants infested with broomrape were counted and the diameters were measured with a ruler, with the assumption that the tubercles are perfectly spherical. The treatments consisted of *F. compactum* or *F. compactum* plus cellulase (4 to 20 U ml^−1^). This work was carried out under containment.

### 2.4. Determination of the Pectinase Content of the Cellulase

One ml of aqueous fungal cellulase samples (10 units mg^−1^), freshly made for each experiment as a solution containing 10 U ml^−1^, and was checked for pectinase activity. Enzyme blank served as the control. Pectinase activity was measured in a reaction mixture consisting of 533 *μ*l of 1% polygalacturonic acid (pectin), 400 *μ*l of 50 mM sodium acetate buffer at pH 5.0 and 67 *μ*l of the cellulase. The mixture was incubated at 37°C for 10 min as outlined by Tonukari et al. [15]. A 100-*μ*l aliquot of the reaction mixture was mixed with 1.5 ml of 1% 4-hydroxybenzhydrazide (Fluka, fluorescence grade) in 0.5 M NaOH. The mixture was heated at 100°C for 10 min, and cooled on ice water. The experiment was performed three times in six replicate tubes. Each sample was assayed three times. Absorbance was measured at 410 nm against the zero time blank as outlined by Lever [16], using a spectrophotometer with a Versa_max_ tunable microplate reader. Pectinase activity of the cellulase was calculated from a standard curve prepared with a D-galacturonic acid (Sigma). One unit of enzyme forms 1 *μ*mol of galacturonic acid from polygalacturonic acid in 1 min under the conditions of the assay.

### 2.5. Data Collection and Statistical Analyses

Broomrape tubercle deaths were recorded at 24 h intervals after fungal inoculation, for 8 to 11 d. Tubercles were visually scored as healthy (translucent, dense, and intact), infected (diseased), or dead (black and soft). The experiments were performed twice, with three replications per treatment. Data are presented for the tubercles present at the time of inoculation. Values are means and standard errors of the means obtained using SAS statistical package [17].

## 3. Results and Discussion

### 3.1. Pectinase Enhances Infection of Broomrape Tubercles by *F. compactum*

**Figure 1:**
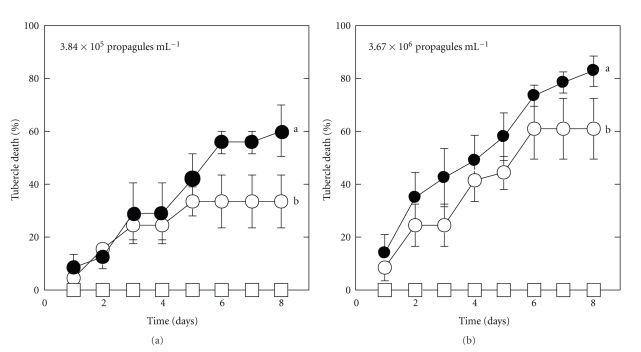
Pectinase enhanced *F. compactum* infection of broomrape growing on tomato roots. Correlation between tubercle mortality of broomrape and fungal inoculum level with • or without ∘ pectinase (a) 3.84 × 10^5^; (b) 3.67 × 10^6^ propagules ml^−1^. Tubercle mortality is represented by percentage of dead tubercles (dark brown, soft to touch, rotten) compared to the total number of tubercles present on plant roots at the time of inoculation. The trial was conducted twice, each treatment with three replications.

**Figure 2:**
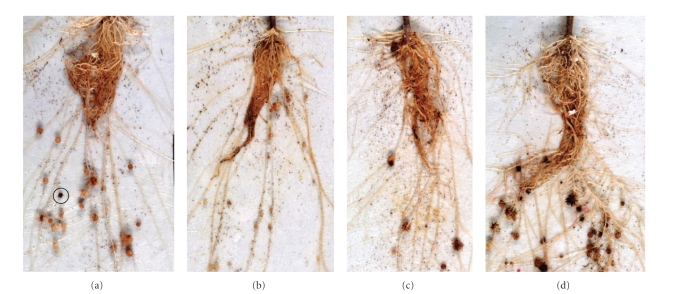
Broomrape tubercles attached to tomato roots showing pectinase enhanced virulence for *F. compactum*. The circle indicates a brown tubercle which was present before inoculation. Each plant bore an average of 16 ± 3 live tubercles. The pictures are from same experiment. *F. compactum* inoculum level was 3.67 × 10^6^ propagules ml^−1^ per inoculated bag.

Experiments were performed to ascertain whether pectinase could accelerate fungal infection of broomrape tubercles. The levels of infection caused by *F. compactum* plus pectinase in both experiments were always better than those achieved by the fungus without pectinase (Figures 1(a) and 1(b)). The numbers of broomrape tubercles infected continuously increased over time throughout the period of observation. This was not only due to inoculum buildup in the tomato root system but also to the enzyme action. This study wanted to ascertain whether the added pectinase would have an effect on suboptimal *F. compactum* inoculum [14]. Therefore, subthreshold levels of inoculum (≤10^6^ propagules ml^−1^) were investigated in the presence of pectinase. Pectinase alone had no effect on the tubercles. *F. compactum* alone killed 30% of broomrape tubercles at the suboptimal inoculum levels (Figure 1(a)). At the lowest inoculation density, the addition of pectinase caused a more rapid response to the fungus. *F. compactum* (3.84 × 10^5^ propagules ml^−1^) combined with 11 U ml^−1^ pectinase killed more broomrape tubercles than the *F. compactum* alone (Figures 1(a) and 1(b)). The killing of broomrape tubercles indicated that pectinase enhanced the virulence of *F. compactum* (Figure 2).

### 3.2. Infection of Broomrape Tubercles by *F. compactum* Plus Mixed Enzymes

**Figure 3:**
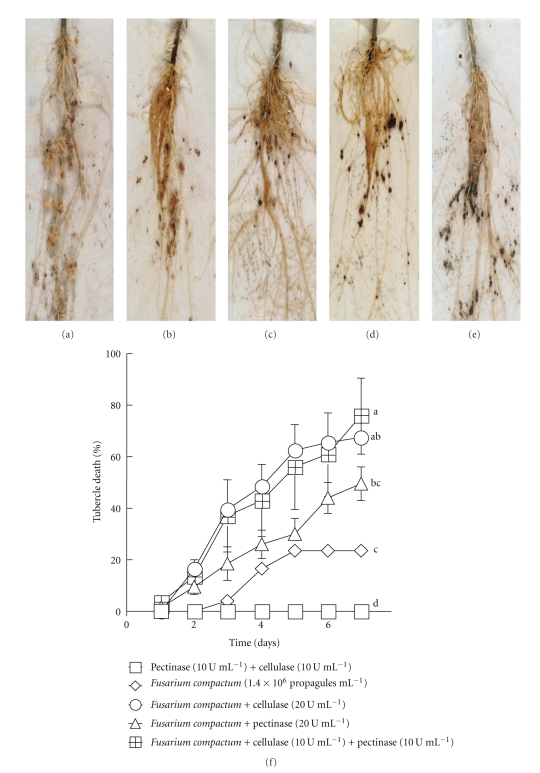
(a)–(e) Adding pectinase and cellulase to chopped *F. compactum* mycelia enhanced fungal virulence on broomrape. Roots of tomato plants infested with broomrape were sprayed to run-off with 10 ml of 10^6^ propagules ml^−1^ of chopped mycelia in 0.01% Tween 20. (a) A total of 20 U solutions of pectinase and cellulase (1 : 1) were used as a control treatment. (b) 1.4 × 10^6^ propagules ml^−1^ homogenized mycelia alone, (c) mycelia plus 20 U pectinase, (d) mycelia plus 20 U cellulase, and (e) 10 U pectinase and 10 U cellulase (20 U total enzyme)  ml^−1^ of homogenized mycelial suspension. Each plant bore an average of 35 ± 6 healthy tubercles at inoculation. (f) The graphical representation of (a) to (e). □ Pectinase (10 U ml^−1^) plus cellulase (10 U ml^−1^), *◊F. compactum* (1.4 × 10^6^ propagules ml^−1^), ∘ *F. compactum* plus cellulase (20 U ml^−1^), ∆ *F. compactum* plus pectinase (20 U ml^−1^), *⊞F. compactum* plus cellulase (10 U ml^−1^) and pectinase (10 U ml^−1^).

Since both enzymes separately enhanced the severity of tubercle infection, it was ascertained whether the pathogenicity of *F. compactum* (1.4 × 10^6^ propagules ml^−1^) on broomrape tubercles could be synergistic through the joint action of the enzymes (10 U ml^−1^ of each enzyme) (Figure 3). The broomrape tubercles on the tomato roots were large and healthy in the absence of fungal treatment but, at times, a few naturally brown tubercles could be seen (Figure 3(a)). *F. compactum* alone infected all the inoculated tubercles but did not kill any significant number (Figure 3(b)). Infested tomato roots that were inoculated with mycelia plus pectinase (20 U ml^−1^) (Figure 3(c)) had over 50% tubercles dead one week after treatment. Those inoculated with mycelia plus cellulase (20 U ml^−1^) (Figure 3(d)) had above 60% mortality. A mixture of both enzymes with *F. compactum* increased fungal infection of broomrape by *F. compactum* (Figure 3(e)). The response of the infected tubercles varied from 100% kill to mild infection, depending on tubercle size. Mycelia plus cellulase (20 U ml^−1^) mix provoked about the same level of infection as mycelia in solution with cellulase and pectinase (10 U ml^−1^ of each enzyme) (Figure 3(f)).

### 3.3. Mixed Enzymes Effect on Broomrape

**Figure 4:**
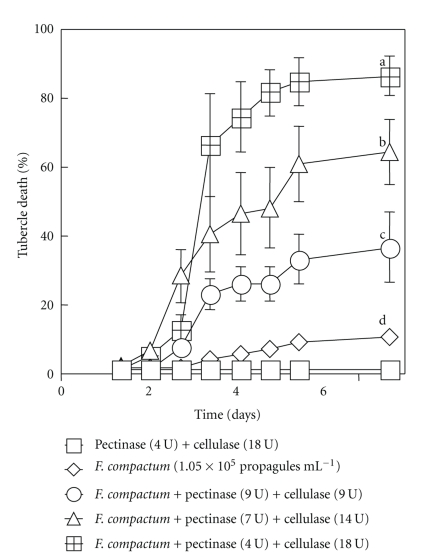
Strong increase in virulence stimulated by cellulase with some amount of pectinase when the fungus is at suboptimal levels. Roots of tomato plants infested with broomrape were sprayed with *◊* 1.05 × 10^5^ propagules ml^−1^, plus ∘ 9 U pectinase and 9 U cellulase, ∆ 7 U pectinase and 14 U cellulase or *⊞* 4 U pectinase and 18 U cellulase per ml of homogenized mycelial suspension. The controls **□**, which all gave the same response were water alone, and pectinase plus cellulase (4 U : 18 U ml^−1^) aqueous solution alone.

It is logical to think collective mixing of pectinase and cellulase to *F. compactum* will effectively enhance the fungal biocontrol potential: thus, it was tested. At various enzyme compositions, the actions of pectinase and/or cellulase enhanced the virulence of *F. compactum* on broomrape. The *F. compactum* treatment (1.05 × 10^5^ propagules ml^−1^) that caused only a hypersensitive reaction (9% death) on the tubercles caused about 35 to 85% tubercle death when the tubercles were treated with mixtures that contained mycelia, pectinase, and cellulase in various ratios (Figure 4). Higher tubercle infection by *F. compactum* was observed with a high ratio of cellulase to pectinase. Beginning from day 4 after *F. compactum* inoculation on broomrape infested tomato plants; statistically significant differences among treatments were observed (Figure 4). Broomrape infested roots coinoculated with chopped mycelia plus pectinase and cellulase had substantial tubercle infection and subsequent large numbers of dead tubercles (35–85%) (Figure 4). This system ensures easy examination of seedling attachment to the tomato roots and easy observation of tubercle infection in the course of the experiment.

### 3.4. Pectinase Content of the Cellulase

The activity catalyzed by an enzyme is a measure of the amount of enzyme present. It was observed that cellulase seems to overshadow pectinase in the spray mix. Thus, it was necessary to ascertain whether the cellulase is pure. Values pooled from three sets of experiment showed that in a 1 mg (10 U mg^−1^) cellulase preparation, 0.055 mg pectinase (1.1 U mg^−1^) is present. This means the 10 U cellulase has a 0.06 U of detectable pectinase. This, however, is not statistically significantly different from the blank.

 The basis for this study stems from previous studies [13] which examines single enzyme-assisted effect on fungi pathogenicity. The *Orobanche* were at three main phases of their life cycle (seed, germination, and parasitic phases) at fungal inoculation. Tubercles are usually present at the germination phase and later become parasitic. In this study, the tubercles were mostly at the germination phase. In apparently healthy tubercles, it may be possible to find diseased tubercles (as depicted in Figure 2); such tubercles deaths were induced by accidental infestation. In total, these results demonstrate that exogenous pectinase as well as cellulase can contribute to pathogenicity. The mycelia plus enzyme mix killed some broomrape tubercles, thus they could not form new seeds to replenish the *Orobanche* seed bank. The rationale of approach is also applicable to soil grown tubercles where the presence of enzyme may be exogenous. The polyethylene bags maintain an environment with constant film of moisture in the system. This is well taken care of in nature because the tubercles are borne on the roots and roots are mostly below the soil surface. The temperature in the growth chamber was kept at 25°C to induce infection by *F. compactum.*

As demonstrated in this study, *F. compactum* was assisted in macerating parenchymatous tissue of broomrape tubercles. Descriptions of actions of cell wall degrading enzymes are given by Wanjiru et al. [11]. *F. compactum* can infect the tubercle by degrading the cell wall components and invading the tissues and cells. Pectinolytic and cellulolytic activities of the enzymes (in this case of fungal origin) result in a loss of structural integrity in the tubercle and characteristic damage. Symptom development was accelerated by probably tubercle cell expansion by the enzymes. The enzyme substrate range and mechanism of action could partly explain the interaction between *F. compactum* and the enzymes on broomrape tubercles. The pathogenicity of *F. compactum* plus the enzyme mix observed could rarely be observed without pectinase and cellulase at the inoculum level used. Besides, neither pectinase nor cellulase was ordinarily able to injure and kill broomrape at the concentration used (max. 20 U ml^−1^), demonstrating that the broomrape tubercle infection and death observed are due to the addition of a mycoherbicidal organism. The role of pectinase and cellulase in the degradation of cell wall material as observed in this study corresponds with the reports of many authors [10, 11, 18, 19]. In the previous paper, the results of using cellulase were reported [13]. In this experiment, similar results were obtained. The results reported by Sasaki and Nagayama [20] showed that the fungi pathogenicity was not always proportional to the enzyme activity.

A 0.6% pectinase activity was detected in the cellulase preparation. It is known that the level of *β*-glucosidase in an enzyme preparation may affect the result of cellulase assays, for example, for the estimation of activities of extracellular cellulase enzymes produced by *Trichoderma*. As pointed out by Kumpoun and Motomura [21], the pectinase used in their study is mainly pectinase, but some glycosidases were also present. The presence of *β*-glucosidase or other enzymes, such as cellobiose phosphorylase, which are required for cellobiose metabolism and to enhance cellulose hydrolysis but which are not, strictly speaking, cellulases or rhamnosidase and *β*-glucosidase activities in pectinase [22] could further complicates research findings. Care must be taken in interpreting the results of pathogenicity tests as the enzyme preparation may contain a battery of enzymes. This result may suggest the need to study the presence of other enzymes in an enzyme of interest even if the enzyme of interest is from a commercial source. 

 Contrary to some of the findings associated with enhanced *F. compactum* infection on broomrape, *F. oxysporum* pathogenicity on broomrape was not enhanced by pectinase and/or cellulase. Thus, although *F. oxysporum* and *F. compactum* belong to the same genus, they may have different mycoherbicidal mechanisms. Mycelia of *F. compactum* did not penetrate nor show apparent damage to the tomato roots [23] as also observed in this study. In conclusion, pectinolytic and cellulolytic activities are widely exhibited by bacteria and fungi. The enzymatic activities can predispose broomrape tubercles to infection by fungi, in this case, *F. compactum*. The latter fact adds to the commercial value of *F. compactum* as a potential mycoherbicide when sufficiently virulent.

## References

[1] Sauerborn J (1991). *Parasitic Flowering Plants: Ecology and Management*.

[2] Joel DM, Kleifeld Y, Losner-Goshen D, Herzlinger G, Gressel J (1995). Transgenic crops against parasites. *Nature*.

[3] Desouky EM (2007). Production of cellulase by *Penicillium hordei* and pectinase by *Aspergillus ustus* under solid state fermentation conditions. *New Egyptian Journal of Microbiology*.

[4] Willats WGT, Steele-King CG, McCartney L, Orfila C, Marcus SE, Knox JP (2000). Making and using antibody probes to study plant cell walls. *Plant Physiology and Biochemistry*.

[5] Chellapandi P, Jani AA (2009). Enhanced endoglucanase production by soil isolates of Fusarium sp. and Aspergillus sp. through submerged fermentation process. *Turkish Journal of Biochemistry*.

[6] Cianchetta S, Galletti S, Burzi PL, Cerato C (2010). A novel microplate-based screening strategy to assess the cellulolytic potential of trichoderma strains. *Biotechnology and Bioengineering*.

[7] De Castro AM, Pedro KCNR, Da Cruz JC, Ferreira MC, Leite SGF, Pereira Jr. N (2010). *Trichoderma harzianum* IOC-4038: a promising strain for the production of a cellulolytic complex with significant beta-glucosidase activity from sugarcane bagasse cellulignin. *Applied Biochemistry and Biotechnology*.

[8] Linger JG, Adney WS, Darzins A (2010). Heterologous expression and extracellular secretion of cellulolytic enzymes by zymomonas mobilis. *Applied and Environmental Microbiology*.

[9] Morozova VV, Gusakov AV, Andrianov RM, Pravilnikov AG, Osipov DO, Sinitsyn AP (2010). Cellulases of Penicillium verruculosum. *Biotechnology Journal*.

[10] Lebeda A, Luhová L, Sedlářová M, Jančová D (2001). The role of enzymes in plant-fungal pathogens interactions. *Zeitschrift fur Pflanzenkrankheiten und Pflanzenschutz*.

[11] Wanjiru WM, Zhensheng K, Buchenauer H (2002). Importance of cell wall degrading enzymes produced by *Fusarium graminearum* during infection of wheat heads. *European Journal of Plant Pathology*.

[12] Babalola OO (2007). Pectinase and cellulase enhance the control of *Abutilon theophrasti* by *Colletotrichum coccodes*. *Biocontrol Science and Technology*.

[13] Babalola OO (2010). Exogenous cellulase contributes to mycoherbicidal activity of *Fusarium arthrosporioides* on *Orobanche aegyptiaca*. *International Journal of Agronomy*.

[14] Amsellem Z, Kleifeld Y, Kerenyi Z, Hornok L, Goldwasser Y, Gressel J (2001). Isolation, identification, and activity of mycoherbicidal pathogens from juvenile broomrape plants. *Biological Control*.

[15] Tonukari NJ, Scott-Craig JS, Walton JD (2000). The *Cochliobolus carbonum* SNF1 gene is required for cell wall-degrading enzyme expression and virulence on maize. *Plant Cell*.

[16] Lever M (1972). A new reaction for colorimetric determination of carbohydrates. *Analytical Biochemistry*.

[17] SAS 2004. SAS 9.1 Companion for windows.

[18] Chanliaud E, De Silva J, Strongitharm B, Jeronimidis G, Gidley MJ (2004). Mechanical effects of plant cell wall enzymes on cellulose/xyloglucan composites. *Plant Journal*.

[19] Esquerré-Tugayé MT, Boudart G, Dumas B (2000). Cell wall degrading enzymes, inhibitory proteins, and oligosaccharides participate in the molecular dialogue between plants and pathogens. *Plant Physiology and Biochemistry*.

[20] Sasaki I, Nagayama H (1995). B-glucosidase of *Botrytis cinerea*: its involvement in the pathogenicity of this fungus. *Bioscience, Biotechnology and Biochemistry*.

[21] Kumpoun W, Motomura Y (2001). Degradation of pectic polysaccharides in various fruits by pectinase derived from *Aspergillus niger*. *Bulletin of the Faculty of Agriculture and Life Science Hirosaki University*.

[22] Mandalari G, Bennett RN, Kirby AR (2006). Enzymatic hydrolysis of flavonoids and pectic oligosaccharides from Bergamot (Citrus bergamia Risso) peel. *Journal of Agricultural and Food Chemistry*.

[23] Cohen BA, Amsellem Z, Lev-Yadun S, Gressel J (2002). Infection of tubercles of the parasitic weed *Orobanche aegyptiaca* by mycoherbicidal , *Fusarium* species. *Annals of Botany*.

